# Supplementary material for: Prognostic Impact of the HFA-PEFF Score in Patients with Acute Myocardial Infarction and an Intermediate to High HFA-PEFF Score
Source: J Clin Med. 2022 Aug 5;11(15):4589. doi: 10.3390/jcm11154589 (PMC9369752; doi:10.3390/jcm11154589)
Supplement: Supplementary file 1 [file jcm-11-04589-s001.zip › jcm-1823454-supplementary.pdf]

Supplemental materials

Supplemental table S1. Study definitions of individual HFA-PEFF criteria compared with the original HFA-PEFF definition

HFA-PEFF criteria	Study definition	Original HFA-PEFF definition
Major criteria: 2 points		
Functional	1. septal e' < 7 cm/s or lateral e' < 10 cm/s or 2. Average E/e' ≥ 15 or 3. PASP > 35 mmHg)	1. septal e' < 7 cm/s or lateral e' < 10 cm/s or 2. Average E/e' ≥ 15 or 3. TR velocity > 2.8 m/s (PASP > 35 mmHg)
Morphological	LAVI > 34 ml/m2	1. LAVI > 34 ml/m2 or 2. LVMI ≥ 149/122 g/m2 (m/w) and RWT > 0.42
Biomarker (sinus rhythm)	NT-proBNP > 220 pg/ml	NT-proBNP > 220 pg/ml or BNP > 80 pg/ml
Biomarker (atrial fibrillation)	NT-proBNP > 660 pg/ml	NT-proBNP > 660 pg/ml or BNP > 240 pg/ml
Minor criteria: 1 point		
Functional	Average E/e' 9–14	1. Average E/e' 9–14 or 2. GLS < 16%
Morphological	LAVI 29–34 ml/m2	1. . LAVI 29–34 ml/m2 or 2. LVMI > 115/95 g/m2 (m/w) or 3. RWT > 0.42 or 4. LV wall thickness ≥12 mm
Biomarker (sinus rhythm)	NT-proBNP 125–220 pg/ml	NT-proBNP 125–220 pg/ml or BNP 35–80 pg/ml

Biomarker (atrial fibrillation)	NT-proBNP 365–660 pg/ml	NT-proBNP 365–660 pg/ml or BNP 105–240 pg/ml

HFA indicates heart failure association; PASP, pulmonary artery systolic pressure; TR, tricuspid regurgitation; LAVI, left atrial volume index; NT-proBNP, N-terminal pro b-type natriuretic peptide; BNP, brain natriuretic peptide; GLS, global longitudinal strain; LVMI, left ventricular mass index; RWT, relative wall thickness.

Supplemental table S2. Proportion of high HFA-PEFF score group and cumulative risks of death according to registration period divided by tertile

Enrollment time (year)	2004 to 2010 (N=334)	2011 to 2012 (N=367)	2013 to 2014 (N=317)	p-value
High HFA-PEFF score (≥4)	89 (26.6)	120 (32.7)	97 (30.6)	0.211
Cumulative risk of mortality (High vs. Intermediate HFA-PEFF score group)	HR 1.99 (95% CI 1.28-3.09), p=0.002	HR 2.22 (95% CI 1.4-3.53), p<0.001	HR 2.16 (95% CI 1.19-3.93), p=0.012	

Data are presented as the n (%) for categorical variables unless otherwise indicated. The p-values for differences were determined using the ANOVA test and from univariate Cox regression. HFA indicates heart failure association; HR, hazard ratio; CI, confidence interval
